# Miniaturized Continuous-Flow Digital PCR for Clinical-Level Serum Sample Based on the 3D Microfluidics and CMOS Imaging Device

**DOI:** 10.3390/s20092492

**Published:** 2020-04-28

**Authors:** Bin Li, Yuanming Li, Andreas Manz, Wenming Wu

**Affiliations:** 1 State Key Laboratory of Applied Optics, Changchun Institute of Optics, Fine Mechanics and Physics (CIOMP), Chinese Academy of Sciences, Changchun 130033, China; 2 University of Chinese Academy of Sciences (UCAS), Beijing 100049, China; 3 Systems Engineering Department, Saarland University, 66123 Saarbrücken, Germany; 4 Bio Sensor & Materials Group, KIST Europe, 66123 Saarbrücken, Germany

**Keywords:** microfluidics, continuous-flow digital PCR, automated graphics processing homebrew

## Abstract

In recent years, the development of polymerase chain reaction (PCR) technology has focused on digital PCR, which depends on the microfluidics. Based on continuous-flow microfluidic technology, this paper designed a miniaturized digital PCR amplification system, and greatly reduced the area required for microdroplet generation and reaction. The core rod. made of polydimethylsiloxane (PDMS), was combined with the Teflon tube to form 3D microfluidics, which requires only one heating source to form the temperature difference required for gene amplification. Only two 34 g needles can form and transmit micro-droplets in a 4-fold tapered Teflon tube, which is the simplest method to generate digital PCR droplets as far as we know, which allows the microdroplet generation device to be free from dependence on expensive chips. A complementary metal oxide semiconductor (CMOS) camera was used as a detection tool to obtain fluorescence video for the entire loop area or a specified loop area. In addition, we developed a homebrew for automatic image acquisition and processing to realize the function of digital PCR. This technique realizes the analysis of clinical serum samples of hepatitis B virus (HBV) and obtained the same results as real-time quantitative PCR. This system has greatly reduced the size and cost of the entire system, while maintaining a stable response.

## 1. Introduction

With the development of life science and medical testing, it has become increasingly important that faster and more accurate disease detection devices can be used for more people. One of the increasingly obvious trends is the miniaturization, integration, and portability of analytical devices. Among the rapidly developing microanalysis systems, micro total analysis systems (μTAS), first proposed by Manz and Widmer in Switzerland in 1990 [1], are the most vigorous. In μTAS, microfluidic chip systems, also known as lab-on-a-chip, are at the forefront of current development and have the broadest prospects for development.

Polymerase chain reaction (PCR), a method for the enzymatic synthesis of specific DNA in vitro, has been proposed for over 30 years. PCR has developed into the conventional key technology in the field of molecular biology, which has greatly promoted the development of various fields of life sciences. The PCR reaction consists of denaturation, annealing, and extension for one cycle. After several cycles, the target DNA can be massively amplified in a short time. In vitro synthesis and qualitative/semi-quantitative detection technology of traditional PCR have been replaced by quantitative real-time PCR (qPCR) technology and related products, which have higher sensitivity, better specificity, and more accurate quantification [2,3,4,5]. 

Considering its rapid development over the past few decades, qPCR technology has been used for the diagnosis of many diseases [2,4,6,7,8,9]. However, there are many factors that affect the amplification efficiency during PCR amplification. There is no guarantee that the amplification efficiency will remain constant during the reaction. In other words, the amplification efficiency may be different between the work sample and the standard sample. This leads to the cycle threshold (CT), the basis that the quantitative analysis relies on, not being constant. Therefore, the quantification of the qPCR is relative to standard curves, and its accuracy and reproducibility still cannot meet the requirements of the quantitative analysis of molecular biology.

At the end of the 20th century, Vogelstein et al. proposed the concept of digital PCR (dPCR) by dividing a sample into tens to tens of thousands of different reaction units, where each unit contains one or more copies of the target molecule (DNA Template) [3,10,11,12,13,14,15]. The target molecule is subjected to the PCR amplification in each reaction unit and the fluorescence signal of each reaction unit is statistically analyzed after the amplification. Unlike qPCR, digital PCR does not depend on CT values, so it is not affected by the amplification efficiency. At the end of the amplification, the average concentration (content) of each reaction unit can be calculated by direct counting or employing the Poisson distribution formula. With control within 5%, digital PCR can achieve absolute quantitative analysis without the need for a standard sample and standard curve.

At present, digital PCR microfluidic devices can mainly be divided into two types: micro-array droplet dPCR [16,17,18,19,20] and continuous-flow dPCR [21]. The former type is actually a miniaturized conventional PCR amplification. In this technique, the reaction solution is uniformly introduced into the reaction chambers or through-holes in a chip by a microfluidic technique to reaction, and the fluorescence signal of each reaction chamber or through-hole is scanned by a method similar to the detection method of the gene chip to calculate the content of the target sequence. It should be indicated that, since the droplet dPCR technology is relatively mature, it is widely used in digital PCR. Continuous-flow dPCR makes the emulsion encapsulate the micro-droplets, allowing the DNA sample and the reagent to flow continuously through three different constant temperature zones to achieve the purpose of the thermal cycle amplification of DNA fragments. Such PCR systems can realize the function of qPCR or dPCR by changing the size of the microdroplets. This method does not require repeated heating or cooling of the PCR system. The heating and cooling rates are generally not limited by the heat capacity of the PCR system. Therefore, the reaction speed is faster when compared to the micro-array droplet dPCR. 

However, the current continuous-flow PCR has certain defects, as shown in Table 1. Most continuous-flow dPCRs use two or more heating sheets to achieve temperature cycling, where the temperature cycling device is bulky and inconvenient to carry [22,23,24]. Furthermore, the droplet is generated by inject printing or a special chip made of polyetheretherketone (PEEK) [22,23,24]. The device above is complicated in structure. At the same time, a surfactant is applied to prevent the droplets from being fused [22,23,24,25]. In order to realize the quantitative function of the digital PCR, it is necessary to count the number of fluorescent droplets. Most devices use a method similar to flow cytometry to detect the fluorescence signal of a droplet one by one. Then, the ratio of droplets containing the target fluorescence to all droplets is calculated, and the content of the target sequence is detected. The core device of this device is a photomultiplier tube (PMT), but the main drawbacks of the PMT are high voltage demand and complicated structure. Disassembly, assembly, and calibration require a higher professional quality of operators and the detection result of the end point detection may be deviated for reagents that will produce a dimer. Meanwhile, the PMT inspection requires surface treatment of the quartz tube to make the detection more accurate [22,24]. Another detection method is cycle-detection, which allows for the observation and collection of the fluorescent droplet data for each thermal cycle. Currently, each temperature circulating pipe has two fibers connected to an angle, one fiber for illuminating the excitation light, and the other fiber for collecting the fluorescence information [23,26]. However, the thermal cycle system generally has about 45 thermal cycles. Therefore, a very large system is required for detecting the fluorescence signal. On the other hand, the fiber breaks easily and is not suitable for disassembly and transportation.

In order to solve the above problems, a novel digital PCR system was proposed in the present work. Using a combination of a needle and a Teflon tube to create a uniform droplet, the proposed device integrates the droplet formation, collection, and reaction, greatly reduces the volume of the droplet generating device, and eliminates the need for surfactants throughout the experiment. The proposed device is simple and greatly reduces the cost of the experiment. The thermal cycle is a monolithic heating method and the reduction of the heating sheet makes the entire device more compact. Image data acquisition is performed on the entire cycle using a complementary metal oxide semiconductor (CMOS) camera, and the product of each cycle is clearly monitored. However, CMOS does not have an analysis function, so an optical system and the analysis homebrew is indispensable. The number of bright droplets and the total number of droplets in the pipeline were analyzed by the homebrew in accordance with the image taken by the CMOS camera. In order to realize the function of the digital PCR, a newly developed imaging software was employed for data processing.

## 2. Materials and Methods

**Figure 1:**
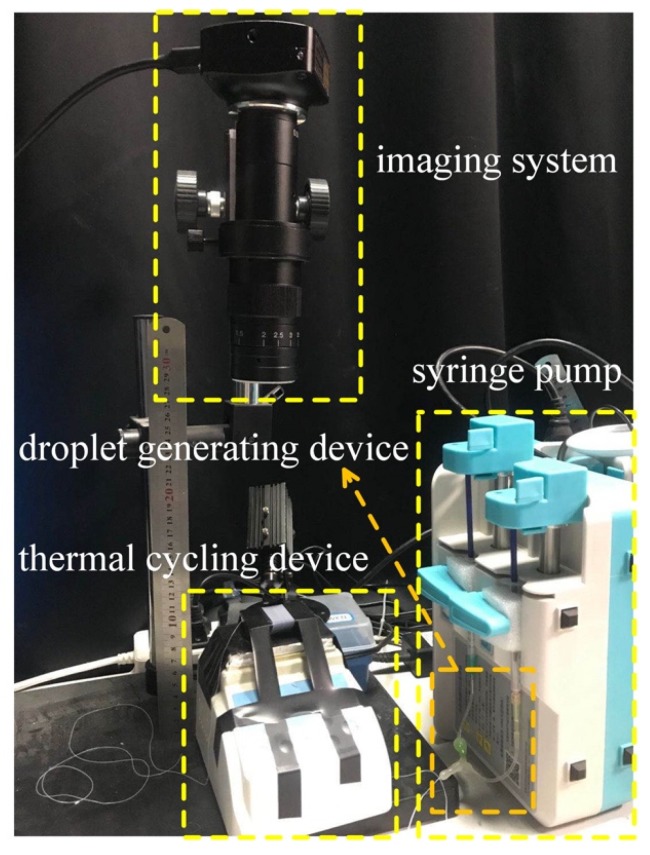
Miniaturized continuous-flow digital polymerase chain reaction (dPCR) system.

The device consisted of four parts: a syringe pump (LD-P2020II, Shanghai, China), droplet generating device comprising two syringes (1 mL, Hongda, Jiangxi, China) and two 34 g needles, a coiled capillary, a heater (JXMINI-80, Shanghai, China) for the thermal cycling, and an imaging system to detect the fluorescent signals (Figure 1). The whole system was about 0.096 m^2^. A 30 cm length ruler is used in the physical drawing to show the size of the whole device more intuitively. 

### 2.1. Droplet Generation

Two 34 g needles were bent into a “V” shape with an angle of about 30 degrees, respectively. A stretched Teflon capillary (OD: 0.3 mm, ID: 0.15 mm, length: 4000 mm) connected two 34 g needles and the other end of the needle is connected to two syringes. The above interfaces were sealed with hot melt adhesive. The syringes contained an water and an oil phase and were mounted on the syringe pumps. The water droplets were entrained in the immiscible oil phase and introduced into the Teflon capillary. Monodisperse droplets of a specified size were produced by controlling the injection speed of the aqueous phase syringe pump. The oil syringe pump was applied to control the oil phase and controlled the flow rate of the droplets. The flow rate ratio of the aqueous phase to oil phase was 1:3. One mL syringes were used in the experiment, and the syringe pumps were adjusted to the specification of 20 mL. The flow rate of the aqueous phase was adjusted to 0.4 mL/h, and the flow rate of the oil phase was adjusted to 1.2 mL/h. It should be indicated that fluoride (3M-novec-7500, Suzhou, China) was used as the carrier liquid in all experiments.

### 2.2. Thermocycling System

The thermocycling system consisted of two parts: a heating station that provided temperature conditions for gene amplification and T-type 3D microfluidics. A 4-m Teflon tube (4-fold stretched) was wound on a quadrangular prism core rod made of a polydimethylsiloxane (PDMS) isosceles trapezoid section to form ladder type 3D microfluidics. The heating pad of the heater was in contact with the upper bottom of the trapezoidal chip to provide the required temperature for the amplification. In order to make the heating uniform, a piece of silicon wafer was placed between the heater and the chip. The upper bottom of the trapezoid in contact with the heater was the high-temperature denaturation zone, while the lower bottom of the trapezoid was the medium-temperature extension zone and the first waist of the trapezoid (where the liquid droplets flow from the upper bottom to the lower bottom) was the low-temperature annealing zone. The capillary was wrapped around the trapezoidal thermal cycle unit for 50 turns. The circulation unit was placed on a small heater, which provided the required temperature for the denaturation, annealing, and extension of the PCR process. For the experiments carried out in the present work, the denaturation temperature was set to 95 °C, while the annealing and extension temperatures were set to 60 °C. The time of each cycle was determined by the diameter of the temperature regulating device and the flow rate of the oil phase carrying the droplets.

### 2.3. Reagents

The reagents we used in the experiment were the hepatitis B virus DNA Quantitative Diagnostic Kit provided by Northeast Pharmaceutical Group Liaoning Bio-Pharmaceutical Co. Ltd. First, the template DNA was extracted by magnetic bead extraction in five steps. Three 1.5-mL centrifugal tubes were labeled as standard references 1–3, and a 400-uL lysis buffer (solution containing guanidine thiocyanate) was added to each tube. Two hundred uL of standard references 1–3 and 10 uL of the magnetic bead suspension (aqueous solution containing nanometer magnetic beads) were added to each tube. After shaking and mixing the mixture for 10 s, it was placed at room temperature for 10 min and then subjected to short spinning. The centrifugal tubes were placed on the magnetic frame for approximately 2 min, and the supernatant was discarded. After standing it for approximately 30 s, the residual liquid was sucked out. Six hundred uL of the rinse buffer (solution containing sodium chloride and ethanol) was added and pipetted 10 times and then subjected to short spinning. The centrifugal tubes were placed on the magnetic frame for approximately 2 min, the supernatant was discarded, and short spinning was performed. The tubes were then placed on the magnetic frame for approximately 2 min again, and the residual liquid was sucked out and placed at room temperature for 2 min. One hundred uL of the eluent buffer with high pH was added. Then, it was placed at room temperature for 2 min after shaking and mixing for 10 s, and the supernatant was aspirated as the template. Then, the reaction liquid mixture was prepared. Fifty uL of the dPCR reaction mixture was prepared in a 200 uL PCR tube. This mixture contained 18 uL of HBV PCR reaction solution, 2 uL of PCR enzyme mixed solution, and 30 uL of the template. Eight uL of the above reaction mixture was poured into the reagent syringe. Thermal cycling was then performed by the designed dPCR system for DNA amplification and detection. The entire cycle was carried out 40 times and the amplification environment temperature was set to 95 °C for 10 seconds and 60 °C for 30 seconds.

### 2.4. Thermocycling System

**Figure 2:**
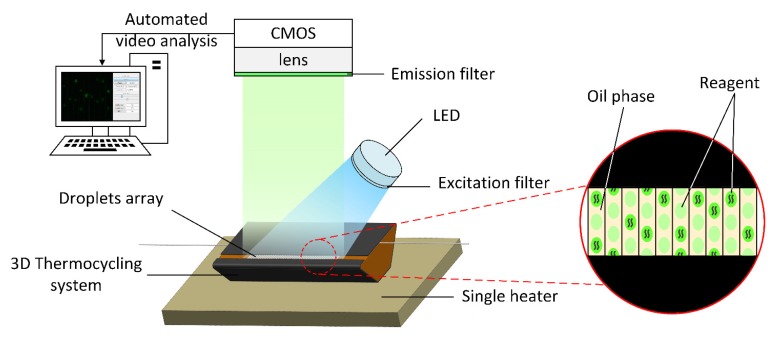
Structure of a miniaturized continuous-flow digital PCR fluorescence detection system.

The fluorescence detection system (Figure 2) employed a high-power LED (Xpe60 W, Cree, NC) with a power of 60 W as the fluorescent excitation source. A Excitation filter (480/30x, Xintian Bori, Beijing, China) was installed in front of the excitation source to remove probable unwanted noises. Moreover, the fluorescence receiving device used a 20-megapixel CMOS camera (complementary metal oxide semiconductor). The front end of the CMOS camera was equipped with an optical system (magnification factor: 300x, working distance: 100–120 mm, CW lens, Shenzhen, China) and an emission filter (520/20 m, Xintian Bori, Beijing, China) to receive the fluorescent signal and the real-time image was displayed on the computer. Since the CMOS camera is equipped with an optical system with adjustable focal length, the structure can monitor the fluorescence of each cycle of the amplification reaction in real time.

**Figure 3:**
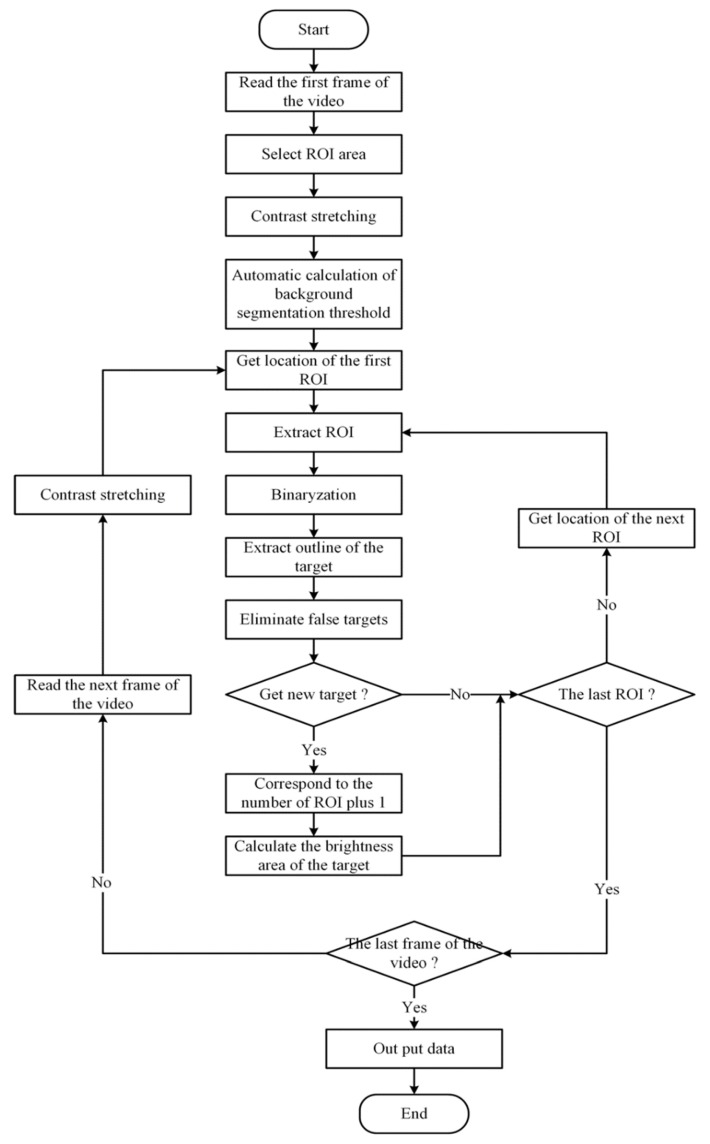
Flow chart of video processing by homebrew.

The video acquired from the CMOS camera was automatically analyzed by homebrew. First, homebrew filters each frame of the video to remove the influence of noise, Then “bright” and “dark” in the video are made more obvious by adjusting the brightness and contrast. After the image was preprocessed, the local area was selected as the analysis area to determine whether there was a bright target. The specific process is shown in Figure 3.

### 2.5. Concentration Calculation

As the target DNA molecules are randomly distributed in the positive reaction units, the real copy number of the target DNA molecules cannot be obtained by directly counting the positive reaction units. Each reaction unit may contain two or more target molecules. We used Poisson probability distribution in Equation (1) to calculate.

(1) $$p=\frac{e^{-\lambda }}{k!}\lambda^{k},\lambda =0,1,2\ldots$$

where λ is the average number of copies of starting DNA molecules contained in each reaction unit and *p* is the probability of each reaction unit containing *k* copy target molecules under certain *λ* conditions. λ is determined by the dilution coefficient m (or number of zones) of the sample solution (i.e., *λ* = *cm*), where c is the original copy number of the sample. When *k* = 0 (i.e., without the target DNA molecule), *P* is the ratio between the number of reaction units with fluorescent signals and the total number of reaction units (i.e., the ratio of negative reaction units). Equation (1) can be simplified as Equation (2):

(2) $$P=1-p(k=0,\;\lambda )=1-e^{-\lambda }=1-e^{-\mathrm{cm}}$$

Through the end-point method, the total number of reaction units n and the number of positive reaction units *f* with a fluorescence signal can be reached, so the proportion of negative reaction units is Equation (3):

(3) $$P=\frac{f}{n}$$

Take the logarithm with e as the base on both sides of the above formula, and get the following equation:

(4) $$cm=-ln(1-\frac{f}{n})$$

When using the method of digital PCR to carry out the absolute quantitative analysis of nucleic acid, only through the proportion of negative reaction units and the dilution coefficient (or partition number) of samples, the average number of nucleic acid copies of reaction units can be determined, thus realizing the accurate quantitative analysis of DNA.

## 3. Results

A CMOS camera was used to record the fluorescence of microdroplets flowing in microchannels. With the increase in the number of thermal cycles, the microdroplets gradually showed two different brightnesses. Microdroplets containing templates pair with free bases through thermal cycling to amplify more target DNA, which makes the fluorescence of the microdroplets brighter. Microdroplets without a template cannot be amplified, so the fluorescence brightness of microdroplets is low.

**Figure 4:**
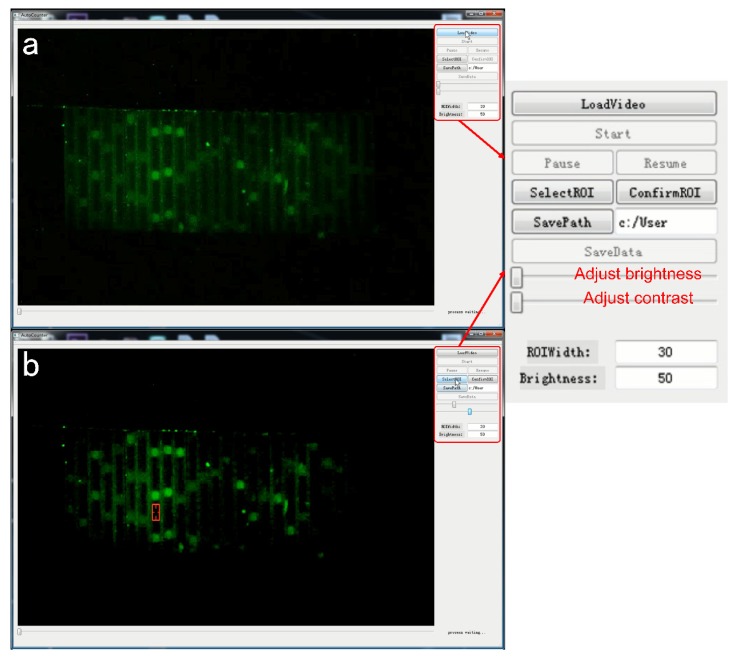
(**a**) Video with unadjusted brightness and contrast. (**b**) Video with adjusted brightness and contrast.

In the initial experiment, we counted the number of droplets from the prerecorded video content of the two brightnesses one by one with our eyes, and then calculated the initial DNA content. However, the time of each reaction was about 30 to 40 min. It takes a lot of time for statisticians to count the number of droplets with their eyes. At the same time, when their eyes look at the display for a long time, it will cause visual fatigue., which will affect the accuracy of the statistical results. Therefore, we designed an automatic fluorescence analysis system. First, the system adjusts the brightness and contrast of the video (Figure 4a) to make the fluorescence more visible. Then, the microdroplets are divided into high brightness and low brightness by comparing the fluorescence brightness of the selected area and counting separately (Figure 4b). As shown in Figure 4, there were many channels in the video. These channels corresponded to the increasing number of thermal cycles from right to left. As the number of thermal cycles increased, the positive droplets gradually became brighter. After about 40 cycles, the brightness of the droplets increased more and more insignificantly, and the reaction entered the plateau phase. The video was imported into this homebrew for contrast and brightness modification, making it easier to distinguish between low-brightness droplets and high-brightness droplets by selecting a pipe in the region that has not reacted. The relative brightness value of the corresponding pixel in the display was automatically recorded in the homebrew, and further applied as the background brightness. The video of the unreacted portion of the reagent was then played, the brightness of the low-intensity droplets was counted, and the data were expressed as a criterion for distinguishing between high brightness and low brightness. Finally, the video after the reaction was completed, the brightness of the droplet was counted again, and output as an EXCEL table. These data were then plotted as dot plots (Movie S1 in Supplementary Materials).

In order to verify that the results obtained by the method in this work were consistent with the ones from the PCR instrument, two sets of repetitive experiments were performed. Three concentrations of reagents were prepared in the first set of experiments. The reagents of each concentration were further divided into two parts to form two sets of parallel experiments. 

**Figure 5:**
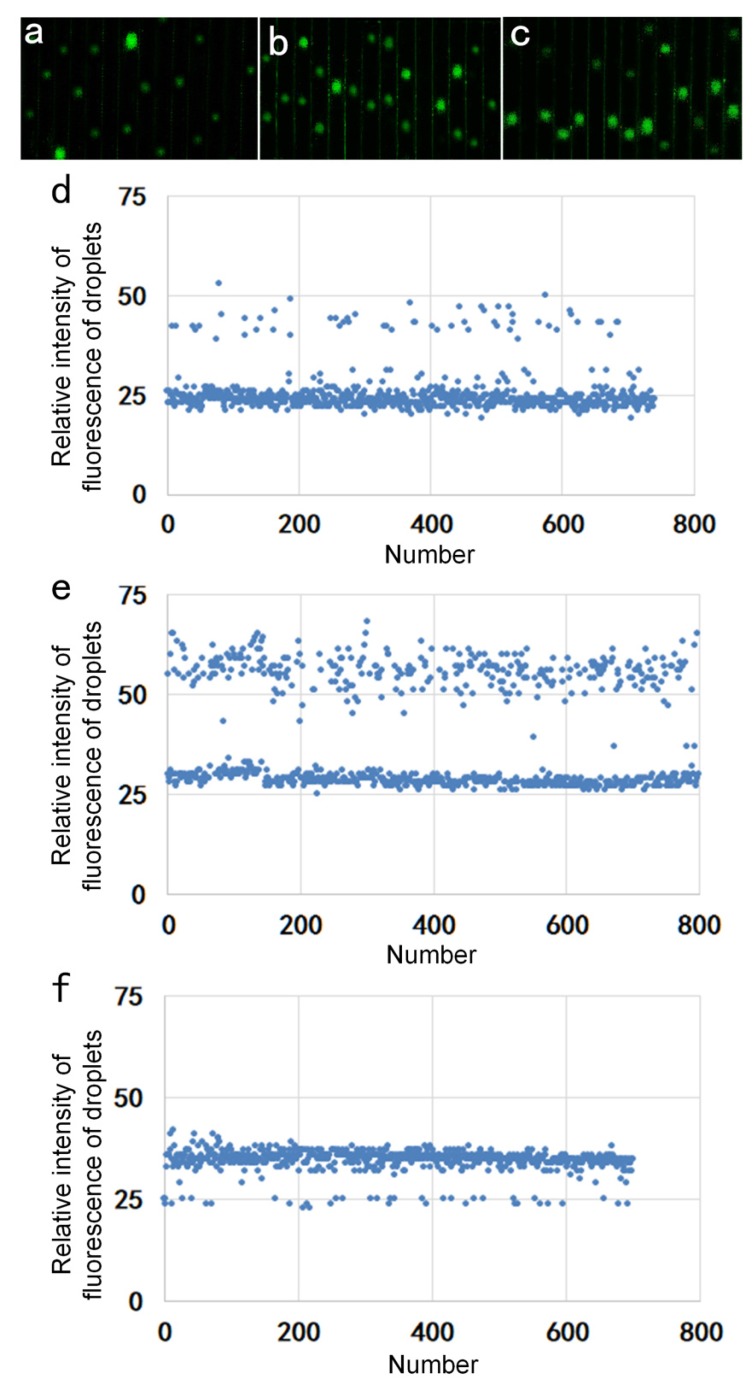
(**a**) Fluorescent image with a reagent concentration of 10^3^ IU/mL. (**b**) Fluorescent image with a reagent concentration of 10^4^ IU/mL. (**c**) Fluorescent image with a reagent concentration of 10^5^ IU/mL. (**d**) Scatter plot of the brightness of the microdroplets with the concentration of 10^3^ IU/mL. (**e**) Scatter plot of the brightness of the microdroplets with the concentration of 10^4^ IU/mL. (**f**) Scatter plot of the brightness of the microdroplets with the concentration of 10^5^ IU/mL.

The standard DNA concentrations in the reagents, entitled by low, medium, and high concentration were 10^3^ IU/mL, 10^4^ IU/mL, and 10^5^ IU/mL, respectively. Three different sample concentrations were sequentially added to the miniaturized continuous-flow digital PCR for the amplification, so that each droplet containing one DNA or no DNA was included. The amplified droplets were illuminated by the excitation light source and exhibited brighter fluorescence. Based on this, Figure 5a–c showed real-time fluorescent images obtained from the CMOS camera. The real-time fluorescence image at low concentration is shown in Figure 5a, and the difference in light and dark droplets can be clearly seen in the figure. It was observed that the droplet distribution was uniform. Figure 5b shows that the number of bright droplets significantly increased when the sample concentration was 10^4^ IU/mL. Figure 5c illustrates that the droplets almost became fully bright as the sample concentration reached 10^5^ IU/mL. Figure 5d–f showed our analysis of the fluorescence signals of reagents with a concentration of 10^3^ IU/mL, 10^4^ IU/mL, and 10^5^ IU/mL, respectively. The scatter plot shows that the positive droplets and negative droplets can be clearly distinguished.

**Figure 6:**
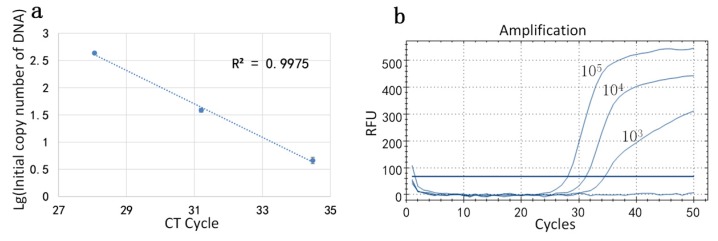
(**a**) Standard curve of reagents measured by our device. (**b**) Amplification curve of the reagents measured by commercial qPCR (BIO-RAD CFX Connect).

After the homebrew counted the droplets, the initial concentration of the reagent was calculated by the formula mentioned in Section 2.5. The standard DNA concentrations were 10^3^, 10^4^, and 10^5^ IU/mL. In this set of experiments, each reagent concentration was divided into three parts and the initial concentration of DNA in the sample was measured by miniaturized continuous-flow digital PCR: The initial concentrations of DNA at low concentration were 416.87 copy/uL, 436.52 copy/uL, and 446.68 copy/uL; the initial concentrations of DNA at medium concentration were 34.67 copy/uL, 39.81 copy/uL, and 40.74 copy/uL; and the initial concentrations of DNA at high concentration were 3.98 copy/uL, 4.68 copy/uL, and 5.25 copy/uL. Then, the logarithm of the initial number of the DNA was taken as the ordinate and the CT value in the amplification curve, generated by the conventional PCR instrument was plotted as the abscissa. The standard curve (Figure 6a) is presented and the corresponding amplification was performed. The amplification curve is shown in Figure 6b. The correlation coefficient R2 of the standard curve was calculated to be 0.9958.

**Figure 7:**
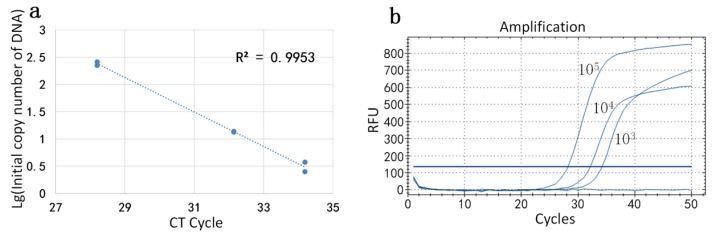
(**a**) Standard curve of the second set of high, medium, and low concentration reagents measured by our device. (**b**) Standard curve of the second set of high, medium, and low concentration reagents measured by commercial qPCR (BIO-RAD CFX Connect).

In order to confirm the stability of our method, we selected different batches of reagents. We used the same experimental procedure as before, and the standard DNA concentrations were still 10^3^, 10^4^, and 10^5^ IU/mL. Each reagent concentration was divided into two parts and carried out by miniaturized continuous-flow digital PCR. Then, the initial concentration of DNA was calculated by the formula. The result showed that the initial concentrations of DNA at low concentration were 138.04 copy/uL and 223.87 copy/uL; the initial concentrations of DNA at medium concentration were 13.49 copy/uL and 13.80 copy/uL; and the initial concentrations of DNA at high concentration were 2.51 copy/uL and 3.72 copy/uL. The coordinate axis drawing method was the same as the first group, and the standard curve is shown in Figure 7a. The result detected by qPCR is shown in Figure 7b. The correlation coefficient R2 of the standard curve was calculated to be 0.9953. The result was consistent with that measured by commercial qPCR, although it was different from those obtained by previous reagents, which also confirms the stability of our system.

## 4. Discussion

At present, the mainstream development direction of new PCR technology is still microfluidic based on chip PCR. After the introduction of the micro total analysis system, microfluidic technology has made it possible to greatly reduce the space required for microdroplet generation and reaction. On the other hand, the reduction in the reaction volume makes the thermal cycle and fluorescence detection more difficult, which are two key processes in achieving absolute quantification in digital PCR. The experiments performed in the present work prove that the droplet generation system, based on miniaturized continuous-flow digital PCR of the CMOS camera imaging device and the 3D microfluidics, can generate droplets of uniform size by using only two 34 g needles without adding any surfactant to the oil, and the droplets are basically so no fusion occurs. The new 3D microfluidics was also very well matched with the small heater. The microfluidics temperature could be stabilized within the required range when the heating device was reduced in overall size. The CMOS-based fluorescence detection system also fully met the detection requirements. As the detection system has a large field of view, it can simultaneously monitor the fluorescence of multiple cycles, which also largely avoids the fluorescence caused by the presence of dimers in the late cycle. The problem of detecting data failures can also serve as a reagent for screening dimers in the assay. Compared to previous reports, our system greatly reduced the size and cost of the entire system while maintaining a stable response. This kind of equipment has a wide application in the fields of biology and medical treatment.

## Figures and Tables

**Table 1:** Comparison of the four droplet dPCRs.

Target	Droplet	Sensing	Chip Material	Chip Channel Modification	Surfactant	The Number of Heaters	Quantification	Ref
Experiment (standardized plasmid clones and environmental samples)	Inkjet printing	Terminal-detection	Silica capillary	Yes	Required	Three	Absolute quantification	[24] ^1^
Clinical (LunX mRNA)	Microchip	Cycle-detection	FEP tubing	No	Required	Two	Relative quantification	[23] ^2^
Experiment (DNA extracted from Caski cells)	HPLC	Terminal-detection	Teflon capillary	Yes	Required	Two	Absolute quantification	[22] ^2^
Clinical (Hepatitis B Virus DNA)	34 g needles	Cycle-detection	Teflon + Silica capillary	No	Free	One	Absolute quantification	This work

^1^ Analytical Chemistry. ^2^ Lab-on-a-chip.

## Supplementary Materials

The following are available online at https://www.mdpi.com/1424-8220/20/9/2492/s1, Movie S1: Counting process of the droplet analysis software.
