# Supplementary material for: Miniaturized Continuous-Flow Digital PCR for Clinical-Level Serum Sample Based on the 3D Microfluidics and CMOS Imaging Device
Source: Sensors (Basel). 2020 Apr 28;20(9):2492. doi: 10.3390/s20092492 (PMC7250024; doi:10.3390/s20092492)
Supplement: Supplementary file 1 [file sensors-20-02492-s001.zip › supplemental data.docx]

Supplemental Data：

Movie S1. Counting process of droplet analysis software

The area with no droplets in the pipeline is selected as the data reading area. At this time, the brightness value of the current area is recorded as the background brightness by the software. Adjust the brightness and contrast of the video to make the droplets clearer and set the brightness criteria for light and dark droplets. After the video starts playing, the droplets in the pipeline begin to flow, and the droplets flowing through the selected area are captured by the software and counted.
